# Mediastinal abscess revealed by computed tomography after pharyngeal fish-bone impaction

**DOI:** 10.1016/j.radcr.2022.09.012

**Published:** 2022-09-26

**Authors:** Julien W. Hsieh, Nicolas Dulguerov, Maxime Mermod

**Affiliations:** Department of Otorhinolaryngology- Head and Neck Surgery, Geneva University Hospitals, 4 rue Gabrielle-Perret-Gentil, CH-1211 Geneva 14, Switzerland

**Keywords:** Foreign bodies, Esophagus, Endoscopy, Sepsis, Abscess

## Abstract

Fishbone impactions in the upper aerodigestive tract are frequent but rarely cause serious complications when recognized and treated early. In this report, we describe the case of a patient that sought medical attention as late as 2 weeks after the fishbone impaction. A 52-year-old male was presented with fever, odynophagia and a toxic appearance. CT scan revealed a large cervicomediastinal abscess. The patient was immediately started on large-spectrum antibiotics, treated by surgical drainage, and recovered uneventfully. This case report highlights the occurrence of severe complications of upper digestive tract fishbone impaction and the usefulness of a preoperative CT scanner in this context.

## Introduction

Impaction of foreign bodies in the upper aerodigestive tract are frequently seen in situations where removal can easily be achieved by identification and removal using esophagoscopy [1]. On the contrary, delayed recognition of foreign body impaction can lead to perforation of the hypopharynx and cervical esophagus. The latter is associated with life-threatening conditions and a high mortality rate due to the development of mediastinitis, big vessels pseudoaneurysm, pleural empyema, septic shock, and death [2]. In this context, a high index of suspicion is warranted to detect those complications. In this case report, we highlight the importance of prompt identification of mediastinal abscess following delayed identification of an impacted fishbone by computed tomography.

## Case report

**Fig. 1:**
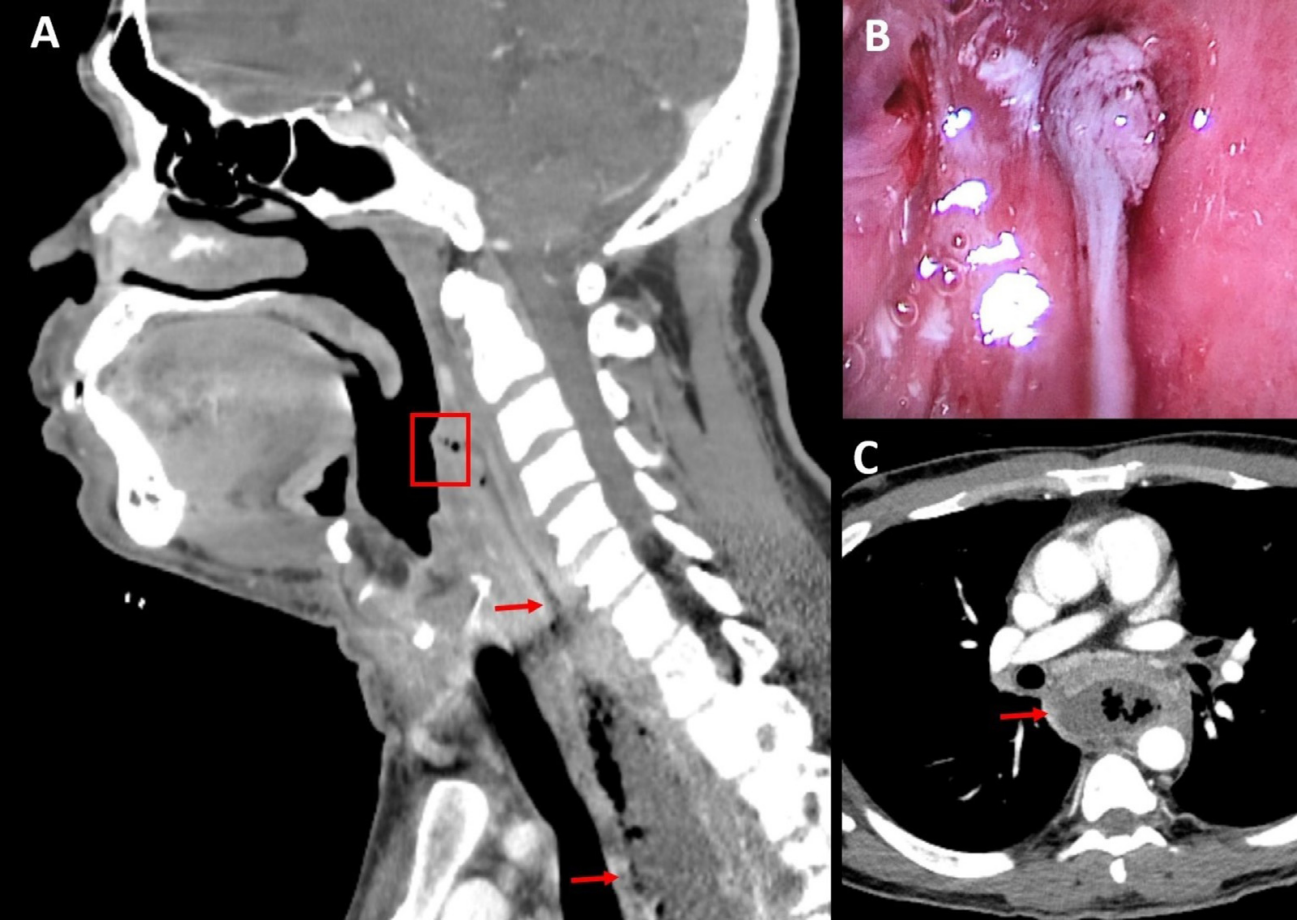
Case illustration by CT scan and endoscopic images (A) Contrast enhanced CT scan in sagittal plane demonstrating the descending mediastinitis (red arrow) with the suspected entry route (red square) (B) Endosopic view of the oropharynx showing the suspected entry route with an inflamed mucosal bud. (C) Contrast-enhanced CT scan in axial plane demonstrating descending mediastinitis (red arrow).

A 52-year-old male presented to the emergency department with odynophagia, retrosternal pain, weight loss, high-grade fever, and tachycardia. Odynophagia started immediately following eating an emperor fish. However, the patient had not sought medical attention. Physical examination showed a right neck swelling and bulging of the posterior and right lateral pharyngeal wall. Blood work showed leukocytosis (13.4 G/L) and an elevated C-reactive protein (166 mg/L). Contrast-enhanced computed tomography scan showed a large abscess extending from the retropharyngeal, right visceral and submandibular spaces to the danger space from C3 to T11 (Fig. 1). The patient received intravenous piperacillin-tazobactam and fluconazole and underwent immediate drainage of the abscess through a right cervicotomy combined with video-assisted thoracoscopy. Pharyngoscopy and oesophagoscopy revealed the suspected fishbone impaction site on the posterior pharyngeal wall (Fig. 1B), but no fishbone was visualized or recovered. Therefore, we hypothesized that the latter eventually passed through the gastrointestinal tract. After surgery, the patient was admitted to the intensive care unit to undergo septic shock resuscitation. The bacterial culture was positive for *Streptococcus anginosus* Group, *Peptostreptococcus anaerobius* and Peptoniphilus. The antibiotic was switched to intravenous co-amoxiclav. The patient was discharged on postoperative day 19 after a favorable evolution.

## Discussion

This case illustrates a dramatic outcome after a neglected pharyngeal fish bone impaction. Fishbone accounts for the majority of ingested foreign bodies in adults [3]. Frequent impaction sites in the oropharynx are the tonsils, tongue base, valleculae, and pyriform recesses, the tonsils being the most common site [4]. The diagnosis is based on the patient's history, symptoms, and careful examination of the pharynx. Symptoms include foreign body sensation, odynophagia, and retrosternal pain. Endoscopy with general anesthesia is mandatory in case of suspicion of an impacted sharp foreign body in the pharynx or the esophagus. Impaction in the posterior wall of the pharynx is rare but is associated with disastrous outcomes due to its proximity with the danger space. Treatment of danger space abscess entails wide-spectrum intravenous empirical antibiotic therapy and early surgical drainage. This approach shows a favorable outcome in 85% of cases [5].

## Conclusion

A 52 years old male with fishbone-induced perforation of the posterior pharyngeal wall was treated by immediate surgical abscess drainage via cervicotomy combined with video-assisted thoracotomy. Pretreatment cervicothoracic CT-scanner is mandatory for the diagnosis and preoperative assessment of cervicomediastinal complications, in cases of fishbone impaction with delayed medical assessment.

## Patient consent

Informed consent was obtained from the patient for the publication of this case report.
